# A Digitalized Silicon Microgyroscope Based on Embedded FPGA

**DOI:** 10.3390/s121013150

**Published:** 2012-09-27

**Authors:** Dunzhu Xia, Cheng Yu, Yuliang Wang

**Affiliations:** Key Laboratory for Micro-inertial Instruments and Advanced Navigation Technology of the Education Ministry, Southeast University, Nanjing 210096, China; E-Mails: 101010203@seu.edu.cn (C.Y.); yuliangwang2012@163.com (Y.W.)

**Keywords:** digital miniaturization, silicon microgyroscope (SMG), automatic gain control (AGC) loop, software phase-locked loop (SPLL), adaptive step least mean square demodulation (AS-LMSD)

## Abstract

This paper presents a novel digital miniaturization method for a prototype silicon micro-gyroscope (SMG) with the symmetrical and decoupled structure. The schematic blocks of the overall system consist of high precision analog front-end interface, high-speed 18-bit analog to digital convertor, a high-performance core Field Programmable Gate Array (FPGA) chip and other peripherals such as high-speed serial ports for transmitting data. In drive mode, the closed-loop drive circuit are implemented by automatic gain control (AGC) loop and software phase-locked loop (SPLL) based on the Coordinated Rotation Digital Computer (CORDIC) algorithm. Meanwhile, the sense demodulation module based on varying step least mean square demodulation (LMSD) are addressed in detail. All kinds of algorithms are simulated by Simulink and DSPbuilder tools, which is in good agreement with the theoretical design. The experimental results have fully demonstrated the stability and flexibility of the system.

## 1. Introduction

The silicon Micro-electromechanical Systems (MEMS) microgyroscope has been developed recently in the automotive industry as a kind of miniaturized angular rate sensor for several applications like rollover detection, inertial navigation, and the electronic stability programs. As is known, it has the merits of small volume, light weight, high reliability and low cost, thus it is easy to digitize and intellectualize and suitable for mass production.

Apart from the micro structure design and vacuum packaging, the readout electronics for this inertial sensors have usually been realized using analog circuit technology by either PCB or ASIC. Considering the investigation on the signal detection and control technology applied in the SMG has significant meaning since it is crucial to improve gyro's accuracy and back-end system integration. Most traditional SMG control and signal detection tasks are implemented only with analog circuits, which are easily susceptible to outer electromagnetic interference with poor device temperature characterization. On the other hand, some intelligent control and signal processing methods can be realized to enhance the stability and flexibility.

Compared with other embedded devices such as Micro-programmed Control Unit (MCU) and Digital Signal Processing (DSP), FPGA is a high performance device integrated with millions of digital logic elements, which can perform more complex numerical computing, logic decision and measuring-control functions even with low power consumption and fast parallel processing. Nowadays, It has been developed into a System on Chip (SoC) integrated device based on a FPGA/DSP hybrid system.

Some mature prototype microgyroscopes have been realized by analog circuit technology [1–3]. However, compared with other digital systems, they obviously lack enough flexibility and compatibility to satisfy different types of gyroscope structures and a variety of applications. Especially, the updated advanced algorithms by changing many circuit parameters cannot be easily performed in a relatively simple analog circuit. In order to further improve the overall system performance, many kinds of digital control circuits have been investigated to date. Though some novel digital systems based on DSP (digital signal processor) have been achieved [4,5], there are some great advantages in using FPGA instead, especially considering its faster parallel speed and lower power consumption, which will greatly better its whole performance eventually. Though a typical digital system based on FPGA was presented in [6], the conversion accuracy was only 12-bit that will inevitably cause much quantization noise and make the signal accuracy poor. In [7,8], a hardware platform for intelligent tuning, model identification and closed-loop operation was developed and tested over a generation two JPL/Boeing pyrex post resonator MEMS gyroscope. The real-time filtering and control of this device was successfully implemented through a digital design based on both a main digital ASIC and a monitoring-function FPGA chip. A software interface allows the user to configure, calibrate, and tune the bias voltages on the microgyroscope. Later on, a more advanced evolutionary computation algorithm for mode-matching tuning was further developed, and also another novel compact Disc Resonator Gyroscope was easily embedded in this compatible digital platform [9,10], which demonstrates the merits of adopting FPGA. Certainly, the JPL gyroscope based on FPGA and 24-bit sigma-delta A/D attained good performance, however, compared with a relatively high resonant frequency, the nearly 50 kHz sampling rate is so low that it may sometimes cause time and phase delay effects. In [11,12], the double closed-loop and force-feedback digital control architectures are implemented with a certain noise and stability performance by using the sigma-delta modulation in both drive and sense modes. In [13,14], with the similar sigma-delta technology, the combination of ASIC and FPGA is implemented to achieve low noise and high Signal Noise Ratio (SNR) capability. Even though the performance is obviously updated by FPGA, for some special applications, we still need to achieve its miniaturization. Based on the previous investigations, here we will offer a digital system based on FPGA with 16-bit accuracy and high processing speeds of up to 300 kHz after miniaturization, which can assure both the system resolution and avoiding processing delays. Meanwhile, all the detailed components, including algorithm simulation and verification in FPGA, will be presented to show the characteristics of our digital system in this work.

## 2. Design of the Hybrid System

**Figure 1.**
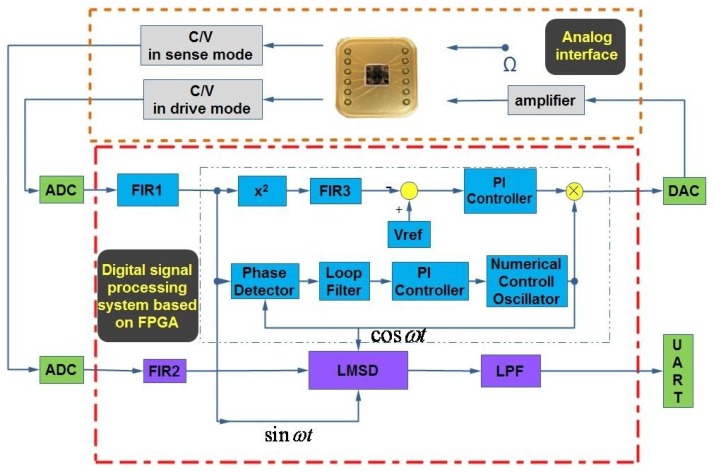
Design scheme of the digital system.

As can be seen in Figure 1, a hybrid detection and control circuitry commonly includes an analog interface circuit and a digital processing system based on FPGA in the drive and sense modes.

The gyroscope structure adopted here is that the drive mode and sense mode are completely symmetric because they are both varying-area style. First, the proof mass is electrostatically driven to single harmonic vibration in drive mode. Then, if the rotation occurs, another single harmonic vibration in sense mode will happen in the perpendicular direction. All the motions can be actuated and sensed through an interdigital combo structure. A detailed schematic has been introduced in our previous work [2]. The whole digital system consists of an analog part and a digital part. For the analog part, there are two capacitance to voltage (C/V) convertors for the output ports of the gyroscope in two modes. One is to detect the varying capacitance in drive mode and the other is specially designed for sense mode. The amplified adjustable feedback AC voltage and constant DC voltage is applied on the input port for driving the proof mass of the gyroscope. For the digital part, there are two off-chip 18-bit A/D convertors and one 16-bit D/A convertor together with their signal conditioning circuits, the Universal Asynchronous Receiver/Transmitter (UART) for transmitting the final demodulated signal, and a high performance core FPGA chip. In FPGA, the automatic gain control (AGC) loop and the software phase-locked loop (SPLL) are realized by an algorithm in drive mode. The automatic gain control (AGC) loop is comprised of an *x*^2^ type amplitude detector, a low pass filter FIR3, a reference voltage *V*_ref_ to set the vibrating amplitude of the gyroscope and PI controller. The software phase-locked loop (SPLL) is comprised of phase detector, loop filter, a PI controller and numerical control oscillator (NCO). Meanwhile, the varying step least mean square demodulation (LMSD) is implemented by algorithm in sense mode. The detailed operation mechanism will be discussed in the following sections.

As for the key analog interface, there have been a lot of papers discussing it extensively. By experimental comparison, the front end with the ring diode detection is adopted to improve the SNR ratio. The minor signal can be picked up by demodulating the Coriolis effect induced aF magnitude of change in the vibrating capacitance using a high frequency carrier up to 10 MHz and precision integrated ring diode HSM2829 (HP company). Almost all the discrete devices like capacitances and resistances with fine temperature coefficients are adopted by the industrial standard.

Different from the full analog circuit design, apart from the necessary front-end, almost other parts are realized in FPGA. Actually, the analog front-end are similarly adopted in drive and sense modes, and the subsequent digital parts include AGC, SPLL and LMSD are all realized using specific Verilog language in FPGA. Here all the 128 order filter FIR and LPF filters are selected from the Megacore IP library embedded in the software development tools. Besides, to save limited hardware resources, 16-bit fixed-point numbers and algorithms are implemented without decreasing the system precision.

### 2.1. AGC Loop in Drive Mode

In the traditional analog electrostatically actuated microgyroscope, the 90 degrees phase difference between driving force and vibrating displacement cannot been ensured in the case of the temperature variation. Therefore, there always exits a certain drift for the natural frequency of microgyroscope even though the self-oscillation is working. To solve this problem, in FPGA a closed-loop self-oscillating driving scheme is utilized. Most discrete device parameters are represented as the numerical values stored in FPGA that can be made immune to outer interferences including temperature drift.

Essentially, the microgyroscope should be a linear sensor only responsive to the input angular rate around the input axis. Hence, the driving status must be stable in both amplitude and frequency. To satisfy these two requirements, closed-loop control must be achieved in the actuation of the microgyroscope. One is that the phase angle of the whole closed-loop *θ* = 2*n*π; the other is that the gain of the whole loop A > 1 at first. The closed-loop actuation of a microgyroscope commonly adopts AGC, which can implement the closed-loop driving with a nonlinear dynamics characteristic, gradually reducing the closed-loop gain of the entire closed-loop to 1 within 100 ms.

**Figure 2.**
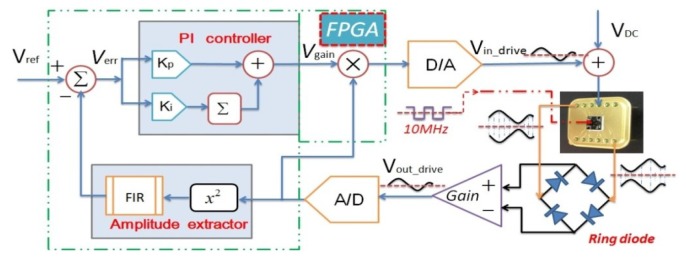
Schematic diagram of digital AGC module.

The principle of the digital AGC module is shown in Figure 2. One 10 MHz carrier signal is directly applied on the proof mass and the ring diode demodulation method is adopted. Here the AGC loop consists of an amplitude extractor and PI controller. In the amplitude extractor, followed by an A/D convertor, the filtered drive detection signal 
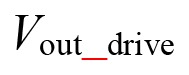
 will be multiplied by itself through an *x*^2^ type amplitude detector to get square term and the DC term. Through another high-order low pass FIR its effective amplitude will then be derived. The error between the actual signal amplitude and expected reference amplitude *V*_ref_ will be calculated. In the PI controller, the error value *V*_err_ will be sent to two branches. One is to calculate the proportional term by multiplying *K*_P_ (proportional coefficient) and the other is to calculate the integral term by multiplying *K*_i_ (integral coefficient) and accumulating the previous results. Next, the key variable gain will be generated using superposition of the proportional term and the integral term. Last, prior to a D/A convertor, the changing drive detection signal 
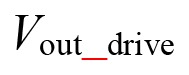
 will be real-time multiplied by the variable gain *V*_gain_ to get an adjustable driving signal 
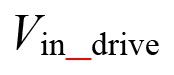
 to push the proof mass of the gyroscope steadily. Once the proportional coefficient and integral coefficient are set properly, the P part will realize the fast tracking and the I part will attain an unbiased effect. Finally, the input signal will always keep constant, *i.e.*, the vibrating amplitude of the gyroscope is forced to be stable even in harsh environment. The PI controller after digitalization can be correspondingly expressed as

(1) $$u(k)=K_{P}\left[e(k)+\frac{T_{o}}{T_{i}}\sum_{i=0}^{k}e(i)\right]$$

(2) $$D(z)=\frac{U(z)}{E(z)}=K_{P}+K_{I}\frac{1}{1-z^{-1}}$$

where *e*(*k*) is the error value at k step, *K_p_* and *K_i_* are proportional and integral coefficients respectively. *U*(*z*) and *E*(*z*) are functions of controller output and error input in z domain. *T_o_* and *T_i_* are sampling and integral time constant respectively.

### 2.2. SPLL Module Based on CORDIC Algorithm

**Figure 3.**
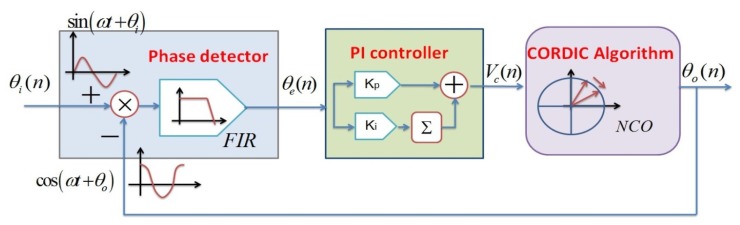
Schematic of the SPLL module.

In drive mode, the driving signal and the displacement detection signal in drive mode are always perpendicular, even though the resonant frequency has a certain drift over temperature. Almost most analog or digital gyroscope circuit systems adopt the PLL technique to sustain the phase difference stability and resonant frequency tracking [15]. In order to realize such self-oscillating in drive mode, software phase-locked loop (SPLL) can achieve this function via software, which makes an output signal track the input signal by the phase difference of 90°. This special algorithm aims to synchronize the output signal with its input signal in frequency as well as in phase. As shown in Figure 3, this adopted SPLL consists of phase detector (PD), loop filter (LF) and numerical controlled oscillator (NCO), which is totally a closed-loop. First, PD is a phase comparator to catch the equivalent phase difference between the output feedback signal *θ*_o_(*n*) and the input signal *θ*_i_(*n*) by multiplication, and then it is filtered by FIR to appropriately generate an error signal *θ*_e_(*n*) for the PI controller input. In the PI controller, *θ*_e_(*n*) is both amplified by the coefficient *K*_p_ and integrated after being multiplied by the coefficient *K*_i_, which is followed by summation. Especially, adjusting here the loop parameter can improve the overall system performance. The output signal *V*_c_(*n*) of PI controller is used to control the frequency and phase of NCO. In fact, NCO is actually a numeric-frequency converter and its output frequency *ω*(*n*) varies with the digital value of control voltage *V*_c_(*n*). In this design, a CORDIC algorithm-based NCO module is adopted, which will simultaneously compute the trigonometric functions such as sine and cosine. Additionally, compared with the traditional look-up table method, this method can save a load of resources and greatly lower the power dissipation in FPGA.

**Figure 4.**
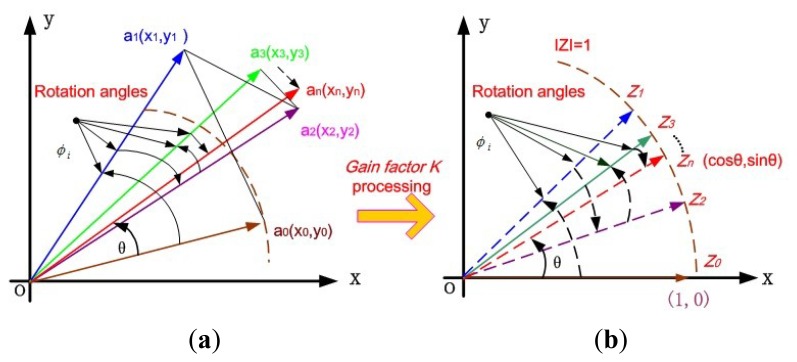
Sketch of the CORDIC algorithm. (**a**) Rotation mode without *K* processing. (**b**) Rotation mode with *K* processing.

Normally in SPLL module, the NCO can be realized by DDS generator [16]. However, it will consume a lot of hardware resources in FPGA due to its non-flexible look-up table method. The Coordinate Rotate Digital Computer (CORDIC) algorithm was first proposed by Volder and Walther in 1959 and has been implemented in fiber optic gyroscopes to lock the laser resonant frequency [17]. Based on the coordinate transformation of the minor angle rotation, the basic idea is to realize the computation of the trigonometric function. Through this algorithm, the final result can be timely calculated after n iterations even over a period of several clocks using addition, subtraction and shift operations, combined with the advanced pipeline technology in FPGA. In Figure 4(a), if an unit vector **a_0_**(x_0_,y_0_) in rectangular coordinate system is rotated to the desired vector **a_n_**(x_n_,y_n_) through n times successive regular rotation operations. After n iterations, sin*θ* and cos*θ* can be obtained, where *θ* is the final total rotation angle. In the case of one time iteration, this vector transformation can be written as:

(3) $$\{\begin{array}{c} x_{i+1}=\cos(\phi_{i})[x_{i}-y_{i}\tan(\phi_{i})]\\ y_{i+1}=\cos(\phi_{i})[y_{i}+x_{i}\tan(\phi_{i})] \end{array}$$

where *φ_i_* is the one-step rotation angle. If we divide a desired rotation *θ* into many steps angles *φ_i_* (*i*=0,1,2,…*n*), *i.e.*, let the limited tan(*φ_i_*) = ±2^−^*^i^*, the rotation-induced production terms tan(*φ_i_*) can be simplified to the convenient digital shift operation in FPGA. Here each step rotation direction can be marked by *d_i_*, then the final rotated angle can be described as

(4) $$\begin{array}{cc} \theta =\sum_{i=0}^{n-1}d_{i}\phi_{i}, & d_{i}\in \left\{-1,1\right\} \end{array}$$

Considering each step rotation angle is a primitive set value, *i.e.*, cos(*φ_i_*) must be a constant. Thus, each step iteration calculation for any rotation angle *θ* can be expressed as

(5) $$\begin{array}{cc} \{\begin{array}{c} x_{i+1}=K_{i}[x_{i}-y_{i}\cdot d_{i}\cdot 2^{-i}]\\ y_{i+1}=K_{i}[y_{i}+x_{i}\cdot d_{i}\cdot 2^{-i}] \end{array} & d_{\text{i}} \end{array}=\pm 1$$

where each step gain factor *K_i_* and general gain factor *K* are given by

(6) $$\{\begin{array}{l} \begin{array}{l} K_{i}=\cos(\arctan(2^{-i}))=1/\sqrt{1+2^{-2i}}\\ K=\prod_{i=0}^{n}K_{i} \end{array} \end{array}$$

In order to realize the shift-add operation, the general gain factor constant *K* can first be extracted from the iteration equations alone, and finally processed as the system uniform gain after all the iterations. Theoretically, when *i* approximates to infinite, *K* will almost equal 0.6037, and conversely the whole system gain approximately equals 1.647. Obviously, the real value of *A_n_* can be calculated by:

(7) $$A_{n}=\prod_{n}\sqrt{1+2^{-2i}}$$

Another auxiliary variable *z_i_* can be used to primitively determine the accumulated angle value and the next step rotation direction by the sign:

(8) $$z_{i+1}=z_{i}-d_{i}\cdot \arctan(2^{-i})$$

In the rotation mode, the final angle accumulator value *z_n_* is just the expected rotation angle *θ*. During the whole iteration process, the rotation direction will be used to decrease the remaining angle value in the angle accumulator. Thus, the final *z_n_* is will infinitely approach to the expected angle value *θ*. By the CORDIC algorithm in rotation mode, the difference function can be simply generalized from Equations (5) and (8) as

(9) $$\left\{ \begin{array}{l} x_{i+1}=x_{i}-y_{i}\cdot d_{i}\cdot 2^{-i}\\ y_{i+1}=y_{i}+x_{i}\cdot d_{i}\cdot 2^{-i}\\ z_{i+1}=z_{i}-d_{i}\cdot \arctan(2^{-i}) \end{array}\right.$$

where

(10) $$d_{i}=\{\begin{array}{cc} -1, & z_{i}<0\\ +1, & \text{else} \end{array}$$

Therefore, the final result in rotation mode can be written as

(11) $$\left\{ \begin{array}{l} x_{n}=A_{n}[x_{0}\cdot \cos z_{0}-y_{0}\cdot \sin z_{0}]\\ y_{n}=A_{n}[y_{0}\cdot \cos z_{0}+x_{0}\cdot \sin z_{0}]\\ z_{n}=\theta ,z_{0}=0 \end{array}\right.$$

where

(12) $$A_{n}=\prod_{n}\sqrt{1+2^{-2i}}$$

Especially, to get the sin*θ* and cos*θ* values, we make full use of the CORDIC algorithm in rotation mode in Figure 4(b). Let the input value y_0_ = 0, x_0_ = 1, then we will find that Equation (11) can be simplified as

(13) $$\left\{ \begin{array}{l} x_{n}=A_{n}\cdot x_{0}\cdot \cos z_{0}\\ y_{n}=A_{n}\cdot x_{0}\cdot \sin z_{0} \end{array}\right.$$

where *y*_0_ = 0, *x*_0_ = 1 and *z*_0_ = 0.

In actual FPGA, by adding, subtracting and shifting operations, the 16-level pipelines structure CORDIC algorithm is easily realized. As for pipeline technology, the shift registers is utilized to insert before each digital adders or subtractors in each level of the module, which aims to avoid the extra time delay in the FPGA implementation of the complex assembly logic circuit. This kind of hardware based on pipeline structure needs total 16 layers of modules, and the inner CORDIC module can be serially cascaded. The shift register in NCO is used to record the corresponding input rotation information of each step. According to this information quadrant, together with the sine and cosine symmetry, the output CORDIC module will be controlled through the control logic module. In this way the input phase range of the CORDIC module can be regulated to ±0.5π, thereby reducing the iteration error and improving the tracking accuracy. Eventually this structure configuration will finally improve the overall system performance.

**Figure 5.**
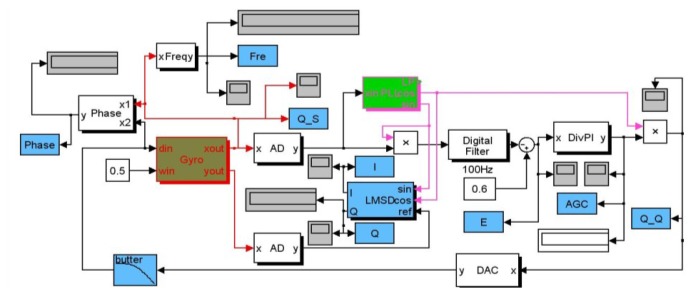
Matlab simulation block diagram.

All simulation blocks in Matlab have been built as shown in Figure 5. The main modules with the abbreviated names such as Gyro, PLL, and LMSD, *etc*. are packaged using internal adjustable parameters. AD and DAC convertor modules are fully realized using programmable function interface with C++ tools.

**Figure 6.**
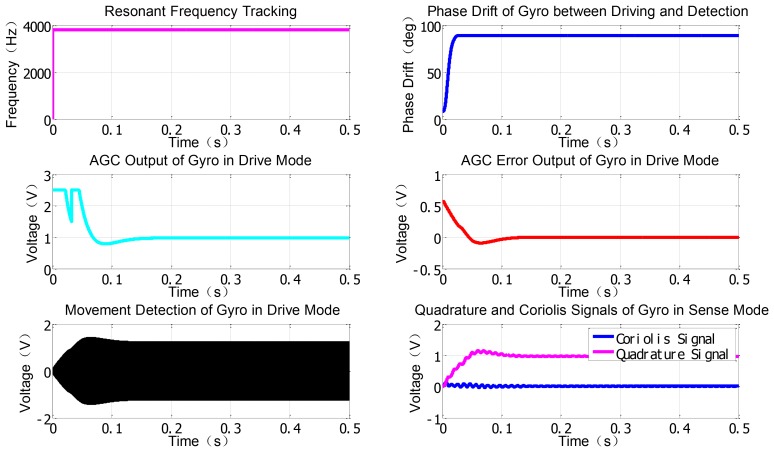
Matlab simulation results.

Other important modules like AGC will be implemented using the discrete multiplier, digital filter and PI modules. Besides, some instruments in gray and specific variables in blue for monitoring the real-time key signal nodes are employed to record some useful curves as shown in Figure 6. From the simulation results in drive mode, the resonant frequency is tracked fast and the phase drift is avoided when the SPLL starts to work. Only after less than 100 ms, the stable AGC control output can perform well and the AGC error rapidly converges to zero. The envelope curves of the resonant movement detection clearly prove that the AGC and SPLL are completely effective. Moreover, the demodulation results of the LSMD are in good agreement with the preset Coriolis and quadrature signals.

**Figure 7.**
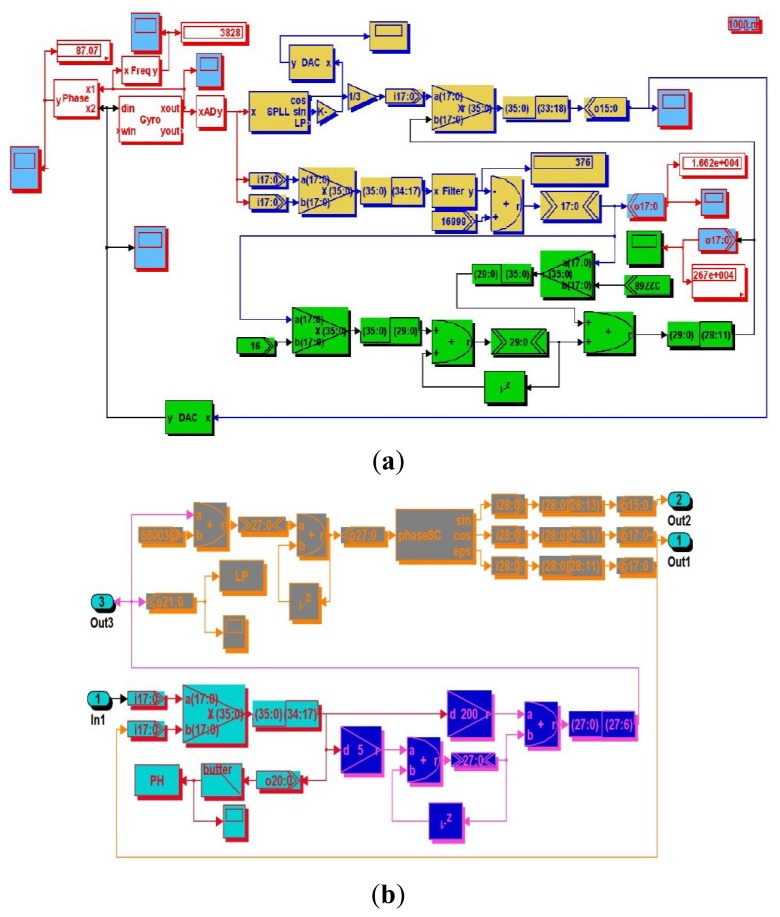
DSPbuilder simulation block diagram based on FPGA. (**a**) The whole system in drive mode. (**b**) Expanded SPLL internal elements.

**Figure 8.**
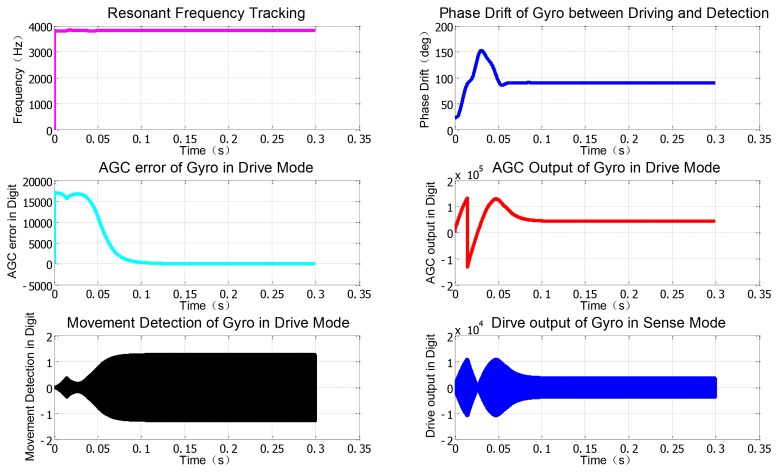
DSPbuilder simulation results with fixed point algorithm.

Similarly, the corresponding simulation blocks created by DSPbuilder tools can be seen in Figure 7, and the simulation results with certain fixed point algorithm in digital domain are given in Figure 8, which are also in good accordance with the former simulation in the analog domain by Matlab. Actually, these two simulation methods are essentially different, the former one is based on the continuous signal expressed by floating numbers for some key nodes' parameters in block diagram, and the latter one is based on the discrete numerical signals expressed by fixed numbers in complement form. It is clearly shown in Figure 7(a,b) that all the effective numerical values in drive mode are expressed in 16-bit binary complement formats, and a lot of binary numbers can been reasonably truncated according to the corresponding precision requirement.

### 2.3. Adaptive Varying Step LMSD Algorithm

**Figure 9.**
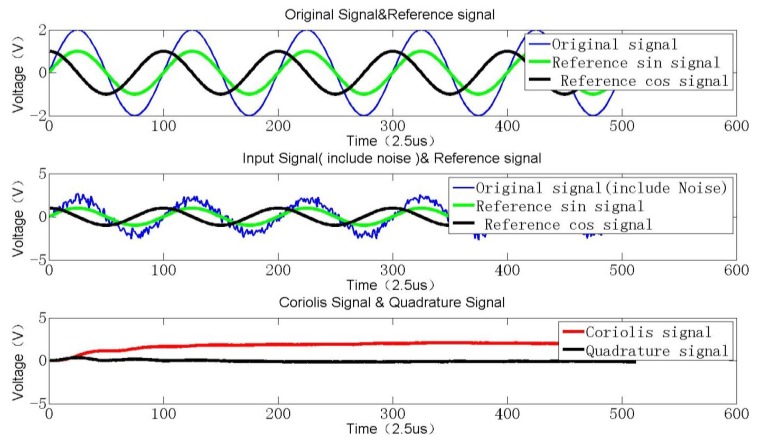
Schematic simulation of LMSD algorithm.

Due to fabrication error induced by the stiffness coupling term between the drive and sense modes, apart from the inevitable noise *n*(*k*), the output signal *S*(*k*) of the SMG essentially can be decomposed into two perpendicular parts. One is Coriolis signal *S_cori_*(*k*) which represents the input rotation angular rate around the input axis, and the other is the quadrature signal *S_qua_*(*k*) which denotes the initial structure-induced error. These two signals are both orthogonal to each other in theory. For an angular rate sensor, the Coriolis signal is utilized to detect the angular rate by synchronous demodulation. However there always exits a slightly vicious double-frequency signal produced by the multiplication operation. Besides, the following high-order LP filter is required especially if the resonant frequency of SMG is rather low, which not only causes a problem of calculation divergence but also consumes more hardware resources. A special demodulation technique using sigma-delta convertor is simulated to evaluate the system performance [18]. Here, to save the limited resources and attain an excellent demodulation effect, a better approach varying step LMSD can be realized to minimize the mean square error between the input signal and the output signal [19]. As shown in Figure 9, a Coriolis signal, *i.e.*, a sinusoidal function, added with an equivalent quadrature signal and some certain noise is simulated as a hybrid input signal. After the adaptive varying step LMSD processing, an expected value of the input signal amplitude can be derived. By comparing this expected signal amplitude *d*(*k*) with the generated input signal ***W***^T^·***r***(*k*), an estimation error *e*(*k*) can be recorded. Through continuously minimizing the variance error, an optimum vector ***W*** will be derived in the end.

(14) $$\min\{E\left[d(k)-W^{T}\cdot r(k)\right]\}$$

(15) $$S(k)=S_{\text{cori}}(k)+S_{\text{qua}}(k)+n(K)$$

(16) $$r(k)=[r_{1}(k),r_{2}(k)]$$

(17) $$e(k)=d(k)-W^{T}\cdot r(k)$$

(18) W(k)=W(k−1)+2u⋅e(k)⋅r(k)

(19) $$A_{\text{cori}}(k)=W_{1}(k),A_{\text{qua}}(k)=W_{2}(k)$$

In Equations (14–19), the varying step LMSD algorithm flow is shown in detail. In Equation (16)***r***(*k*) is actually the reference signal vector with sin(*k*) and cos(*k*) components. In Equation (17), the evaluated ***W***^T^ will converge to a stable vector to make the difference *e*(*k*) between the actual angular rate and the evaluated value ***W***^T^·***r***(*k*) as small as possible. *S*(*k*) denotes the hybrid signals of the coriolis and quadrature signals including the white noise. From Equations (16) to (19), the 4-step iteration will be operated to track the effective amplitudes *A_cori_*(*k*) and *A_qua_*(*k*) of two signals. Especially, *u* is the step length here first set 0.003 and then 0.03 after the error attains a certain range, which can improve the convergence rate.

**Figure 10.**
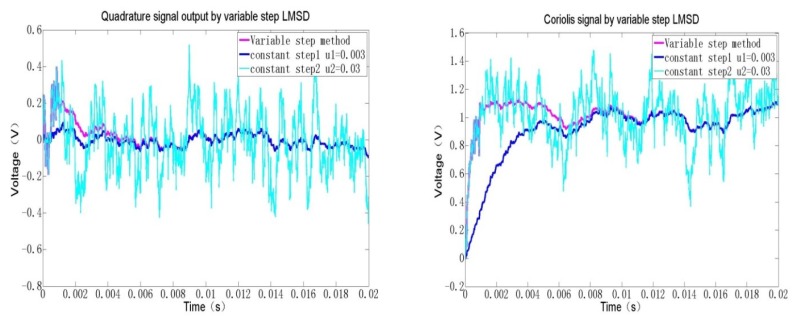
Varying step LMSD effects.

As can be seen from Figure 9, the two direct current terms, which reflect both the Coriolis and quadrature components, can be obtained through a concise iteration algorithm. Obviously, the demodulation results can be rapidly converged to two constant values after 200 loops. The sample rate is high up to 300 kHz, so the interval time is about 33 μs. In actual system, a high-resolution 18-bit A/D converter is selected, therefore, the quantification error in the full range of ±2.5 V is about 0.019 mV, which exceeds far beyond the equivalent angular rate noise. In the FPGA realization, some digital 18-bit multipliers are used to ensure the computation resolution after C/V conversion. Especially the fixed point processing method is adopted to save the limited resources and lower the power consumption. In Figure 10, the Coriolis signal and quadrature signal are calculated by basic LMSD and varying step LMSD methods.

When the step length u is only set to 0.03, the approaching time is short and the divergence magnitude is poor, which demonstrates a strong noise. Whereas if the step length u is only changed to 0.003, the approaching time is longer, but the convergence error becomes smaller than the former. Thus, if we first set u equal 0.03 to improve the approaching rate. After the output signal error approaches a certain range, then we adjust u equal 0.003 to make the divergence error as small as possible. By this intelligent varying step method, we can realize both high-speed and low-noise LMSD method.

## 3. Experimental Results

**Figure 11.**
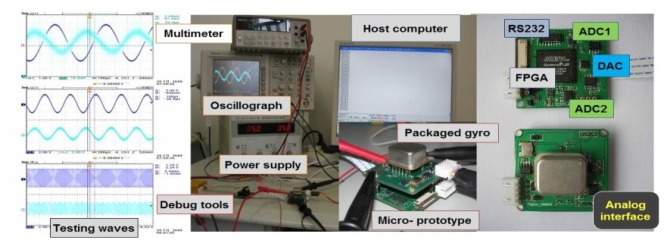
Experimental setup for drive and sense modes.

As can be seen from Figure 11, the digitalized silicon microgyroscope based on an embedded FPGA is implemented with two four-layer circuit boards of 4 cm × 4 cm size. One board is designed for a precision analog interface with demodulated gyroscope signals and the other is for the data converters and FPGA processing. In the specific electronics, two 18-bit resolution ADCs AD7690 (Analog Device) are used to ensure little quantization noises for drive and sense modes. Meanwhile, a 16-bit resolution DAC DAC8832 (Texas Instruments Company) is adopted to ensure the faster sampling rate up to 300 kHz. A high performance FPGA chip EP3C16 (Altera Company) is prefered to satisfy the requirements of the small BGA (Ball Grid Array) package and low power consumption. To facilitate debugging, a special switched JTAG or AS port with 10 pins is attached at the edge of the digital board. Last, the demodulated angular rate signal can be transmitted to the host computer through UART. These components are all low power with high performance.

**Figure 12.**
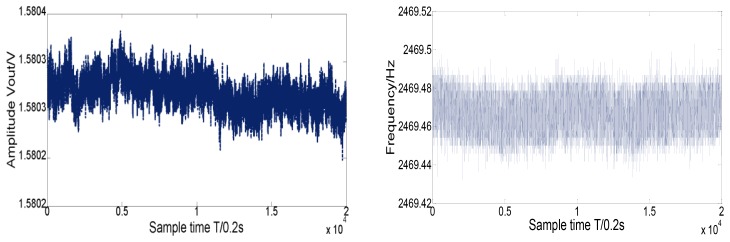
Results of amplitude and frequency outputs in drive mode.

Nearly one hour of detection results of vibrating frequency and amplitude in drive mode are shown in Figure 12. The mean value of the vibrating frequency in drive mode is near 2,469.47 Hz, and its variance is below 0.01 Hz.

**Figure 13.**
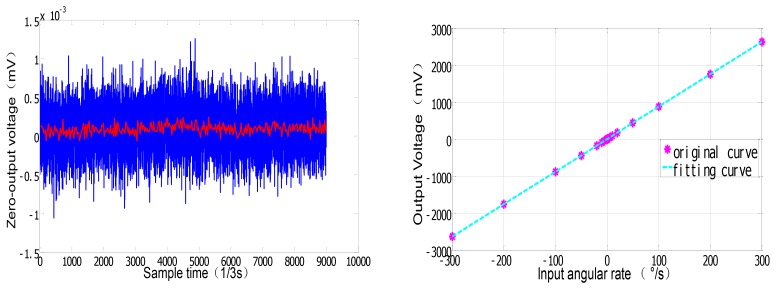
ZRO and scale factor of the microgyroscope at room temperature.

**Figure 14.**
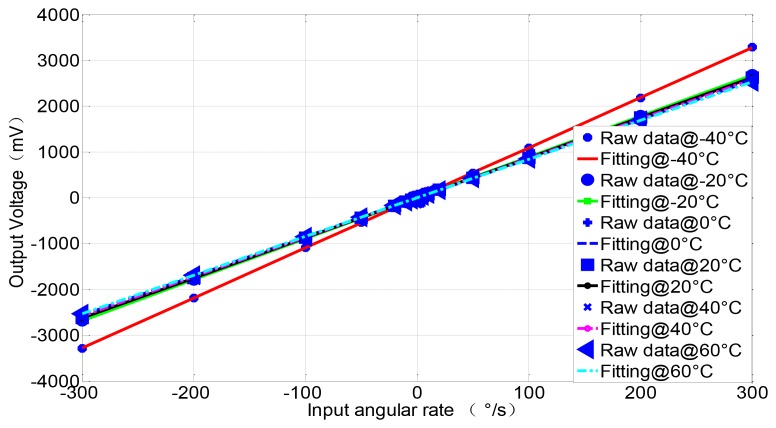
Scale factor of the SMG over temperature.

Similarly, the mean detection voltage value of vibrating amplitude in drive mode is near 1.5803 V, and its variance is 1.45 × 10^−5^ V, thus the stability of both vibrating frequency and amplitude in drive mode have achieved a high precision (the relative error is lower than 1 × 10^−5^). The testing results demonstrate the stability in drive mode and the high precision detection ability of minor signal in sense mode. With the scale factor of 8.775 mV/°/s in full measurement range of ±300 °/s, the nonlinearity is less than 400 ppm and ZRO (Zero Rate output) is very stable at room temperature. Through the overall temperature range from −40 °C to 60 °C, the maximum scale factor deviates by about 10 percent from the room temperature value, which to some extent verifies the temperature stability in harsh environment. All the experimental results can be seen from Figures 12–14.

In Table 1, some key specifications in the experimental results can be compared with that of the previous analog design, where bias stability and linearity are obviously improved in room temperature, noise and the minimum measurable angular rate are decreased by digital filter function, bias stability in full temperature range is improved. According to signal processing theory, the higher the sampling rate is, the better the recovered original signal is. Besides, after we adopt the 300 kHz sampling rate, the phase delay of digitalized signals in each digital modules will be so small that the processing speed can catch up the fast response of the resonant frequency, amplitude and phase of the gyroscope's resonance and the responding Coriolis effect. Thus the gyroscope will work in a fine state and the key specifications shown in Table 1 will naturally be better. If we continue to increase the sampling rate, the specifications will not be improved much, but make the core dynamic thermal power dissipation as high as possible. Regarding these facts, we should select a proper sampling rate as a trade-off.

**Figure 15.**
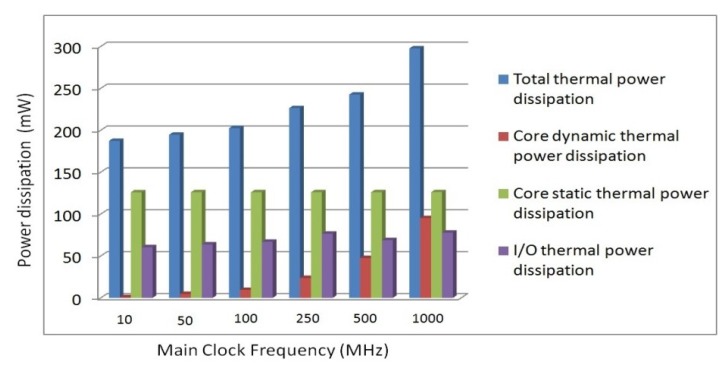
Power dissipation analysis over main clock frequency.

Though the FPGA chip has introduced some more current dissipation than that of the analog design, we still think that it is a good method to realize miniaturization. Along with the analog-digital mixed integrated circuit techniques update, the overall power consumption will be greatly decreased using the new developing low power FPGA chip substitute of 28 nm technology with the compatible package. In Figure 15, the total thermal power dissipation including core dynamic thermal power dissipation, core static thermal power dissipation and I/O dissipation over main clock frequency is illustrated.

When the main clock frequency of more than 100 MHz is adopted, the core dynamic dissipation is increased dramatically, while other thermal power dissipations will not be changed too much. Thus, in order to make the core dynamic thermal power dissipation smaller, only 50 MHz main frequency is adopted in our system. For a lot of clock signals and 300 KHz sampling rate in the digital system, actually it is easy to deploy them by dividing the main frequency.

## 4. Conclusions

The performance of the microgyroscope is greatly decided by the measure and control electronics. From many previous researches, there is a general tendency to select digital technology to replace the traditional analog circuit. Considering the faster parallel speed and low power consumption, a digital microgyroscope based on an embedded FPGA is developed. Different from other FPGA system, the fast sampling rate AD converter with enough 18-bit precision is utilized, and advanced algorithms include SPLL and LSMD are successfully simulated and implemented by hardware programming.

In drive mode, the AGC and SPLL modules work in parallel to ensure the real-time self-exciting and phase locking. The AGC module can make the oscillating amplitude of the proof mass stable in a set constant value. Meanwhile, the SPLL module can track the natural frequency over temperature drift by keeping the 90 degree phase difference between the input driving signal and displacement detection signal. Both the Matlab and DSPbuilder simulations based on FPGA are implemented to validate the algorithm function by adjusting the key parameters, which could provide a reference for the following hardware debugging. In sense mode, to avoid the traditional multiplication method, a varying step LMSD is adopted to realize faster and accurate demodulation. All used algorithms are programmed by fixed point numerical computing, which could save a lot of hardware resources of FPGA and greatly lower the power consumption.

A vacuum packaged microgyroscope is inserted in two pieces of printed circuit boards. The final testing results show that the digital microgyroscope based on embedded FPGA has good performance. Especially, this prototype can be further developed to form a Micro Inertial Measure Unit (MIMU) system by only adding another front-end for another sensing structure without changing the digital parts. Besides, the most previous analog devised are replaced by a digital algorithm, thus our FPGA-based digital gyroscope can have better temperature performance in many harsh applications.

## Figures and Tables

**Table 1.** Performance index comparison of analog with digital microgyro prototype.

**Technical Data**		**Value (Analog)**	**Value (Digital)**
Performance (+25 °C)	*Scale factor*	9.6 mV/°/s	8.775 mV/°/s
	*Bias stability*	15 °/h	12.45 °/h
	*Noise*	0.024 °/s/√Hz	0.01 °/s/√Hz
	*Dynamic range*	±300 °/s	±300 °/s
	*The minimum measurable angular rate*	0.02 °/s	0.01 °/s
	*Linearity*	≤400 ppm	≤200 ppm
Power Supply	*Supply voltage*	±5 V	±5 V
	*Current dissipation*	≤20 mA	≤30 mA
Environment (−40 °C∼+60 °C)	*Bias stability*	3 °/s	1 °/s
	*Temperature drift*	<0.03 °/s/°C	<0.01 °/s/°C
